# Prevalence of and Factors Associated With Depressive Symptoms Among College Students in Wuhan, China During the Normalization Stage of COVID-19 Prevention and Control

**DOI:** 10.3389/fpsyt.2021.742950

**Published:** 2021-10-15

**Authors:** Jincong Yu, Ziyun Yang, Yuqin Wu, Ming Ge, Xuemei Tang, Hongbo Jiang

**Affiliations:** ^1^Education and Counseling Center for Psychological Health, Zhongnan University of Economics and Law, Wuhan, China; ^2^School of Foreign Languages, Zhongnan University of Economics and Law, Wuhan, China; ^3^School of Marxism, Wuhan Railway Vocational College of Technology, Wuhan, China; ^4^Department of Epidemiology and Biostatistics, School of Public Health, Guangdong Pharmaceutical University, Guangzhou, China

**Keywords:** COVID-19, normalization stage, prevention and control, depressive symptoms, associated factor, college students

## Abstract

**Objectives:** The 2019 coronavirus disease (COVID-19) epidemic has led to persistent negative psychological effects on the general public, especially on college students, who are highly susceptible to psychological difficulties, such as fear, anxiety, and depression. Little information is known about depressive symptoms among college students during the normalization stage of COVID-19 prevention and control in China. This study aimed to understand the prevalence of and factors associated with depressive symptoms after a long quarantine time and online learning at home among college students in Wuhan, China.

**Materials and Methods:** A web-based survey was conducted from July to August 2020 during the Chinese summer holiday to collect data on sociodemographic variables, depressive symptoms, and their potential associated factors using an electronic questionnaire among college students in Wuhan, China. The Patient Health Questionnaire-9 (PHQ-9) was used to measure depressive symptoms. Binary logistic regression was used to explore the factors associated with depressive symptoms.

**Results:** A total of 9,383 college students were included in the analysis. The prevalence of depressive symptoms was 15.8% (1,486/9,383) among college students. The binary logistic regression showed that the experience of being quarantined for observation or treatment, family members or friends dying of COVID-19, rarely or never seeking help from others, fewer supportive relatives or friends, less support from family in the past month, a worse relationship with parents at home, a longer time spent daily on electronic devices except for online learning, and feeling anxious in the face of returning to school were independently associated with a higher risk of depressive symptoms. Academic stress and concern about the epidemic were the main reasons for their anxiety.

**Conclusions:** Targeted psychological intervention measures are recommended for college students to improve their mental health during the normalization stage of COVID-19 prevention and control.

## Introduction

The 2019 coronavirus disease (COVID-19) first broke out in China and was declared a pandemic by the World Health Organization (WHO) on March 11, 2020 (1). Globally, as of July 12, 2021, the WHO had reported 186,638,285 confirmed cases of COVID-19, including 4,035,037 deaths (2). To reduce the risk of transmission, strict quarantine measures were implemented to restrict crowd movement nationally (3). However, quarantine is often considered an unpleasant experience and might cause negative psychological effects, including post-traumatic stress symptoms, confusion, anger, anxiety, and depression, which could last for a long time (3, 4).

Apart from the far-reaching influence on the tourist, catering, hotel, and transportation industries, among others (5, 6), the pandemic also had a huge impact on global education, and school closures were usually implemented. To date, 152,692,641 students have been affected worldwide (7). In China, the government also implemented nationwide school closures during the pandemic outbreak (8). The Ministry of Education in China provided suggestions for “suspending classes without suspending learning” in mid-February 2020, which prompted all students to start online learning in the new spring semester (9).

Wuhan, the provincial capital city of Hubei, was the epicenter of the epidemic in China, where COVID-19 was first reported and most cases were confirmed. This city had the largest number (more than 1 million) of college students in China (10). All students attending colleges and universities in Wuhan had to study at home during the spring semester in 2020 due to the epidemic. However, students in other provinces returned to school in April, 2020, when China moved to the normalization stage of COVID-19 prevention and control (11). Delayed opening, refraining from being outdoors, limited interpersonal communication, and long-term online learning might increase student's psychological stress and cause more mental health problems, such as depression and anxiety (12, 13).

Previous studies show that college students have had higher rates of mental health disorders during the initial stage of the COVID-19 epidemic (13–15). The prevalence of college student's depressive symptoms is reported to range from 4.2 to 23.3% in China (13, 15–18) and from 16.1 to 65.8% in other countries (14, 19–22). One meta-analysis conducted by Chang et al. (23) shows that the prevalence of depressive symptoms was 34% among college students worldwide during the COVID-19 epidemic. In addition, some studies have found that living in Hubei Province was a risk factor for mental health problems, including depression and anxiety, among the general population or college students in China during the COVID-19 epidemic (9, 24).

A large number of studies reveal that some factors are associated with individuals' mental health during the COVID-19 epidemic. COVID-19–related experience is a significant factor of concern. A case-control study found that those who were in centralized quarantine had a higher risk of depression (25), and a similar result was found from a cross-sectional study of college students (26). Moreover, having relatives or friends with COVID-19 was associated with a higher risk of depression among college students (15, 27). In particular, those who had relatives or acquaintances who had died of COVID-19 showed a higher suicide risk (28). In addition, social support was recognized as a protective factor against depression. A previous review shows that support from parents was the most important factor protecting against depression in Western children and adolescents (29). During the pandemic, this protective effect of social support on depression was consistently found in college students (15, 22, 30–32). Furthermore, due to the strategy of staying at home, time spent on electronic devices increased markedly (33). Except for online learning, students spent extensive time browsing social media, watching TV, playing games, and so on (34). Increased screen time was demonstrated to be a risk factor for depression in students (35).

In September 2020, when the Chinese fall semester began, all college students returned to Wuhan from different regions of China. Although the pandemic had been almost completely under control in China, sporadic cases or local partial outbreaks remained (32), and confirmed cases continuously entered China from overseas (6, 36), which might increase the student's uncertainty about the pandemic. Therefore, preventing pandemic resurgences was also very important, which would also increase the workload of universities, especially concerning college students' mental health conditions. To our knowledge, most previous studies focus on college student's psychological problems during the initial stage of the COVID-19 pandemic, and little information is available for student's mental health status during the normalization stage of COVID-19 prevention and control in China. Given that college students are highly susceptible to depression, which is a major risk factor for suicide (19, 37), we conducted the present survey to understand the prevalence of and factors associated with depressive symptoms among college students after a long quarantine time and online learning at home and before they returned to universities.

## Materials and Methods

### Participants and Procedure

All students attending the university in Wuhan under the Ministry of Education of the People's Republic of China and majoring in economics, law, and management were recruited. Students at the university came from all regions of China, and almost all lived at home during the survey. Data were collected from July to August 2020 during the Chinese summer holiday. Online questionnaires were distributed to students by the “Yiban System,” which is a comprehensive interactive community integrating education, teaching, life services, and cultural entertainment. All 15,224 non-graduating undergraduate students were asked to participate in the survey by their instructors. All participants took part in the survey voluntarily and anonymously, and they could quit the survey whenever they wanted. Finally, a total of 9,383 students from all 15 schools of the university completed the questionnaire after written informed consent was obtained, yielding a response rate of 61.6%. This study was reviewed and approved by the Institutional Review Board of Zhongnan University of Economics and Law.

### Measures

The questionnaire included three components: sociodemographic variables, including sex, college year, ethnicity, residence and only child status; depressive symptoms (measured by PHQ-9); and a series of potential associated factors (as described below).

### PHQ-9

The PHQ-9 was initially developed for depression screening in primary care settings (38) and has been widely used worldwide (39). Yeung et al. (40) first conducted a validation study of the Chinese version of the PHQ-9 among Chinese Americans. Then, Zhang et al. (41) found that the PHQ-9 had acceptable psychometric properties to screen for depression among Chinese college students. During the COVID-19 epidemic, the scale was also adopted in many studies of college students from China (13, 15, 17, 18) and other countries (19–22). The scale consists of nine diagnostic criteria used to diagnose major depressive disorder based on the Diagnostic and Statistical Manual of Mental Disorders, Fourth Edition (DSM-IV) (38). Participants report the frequency of each symptom during the last 2 weeks. Each item is rated on a four-point scale ranging from zero (not at all) to three (nearly every day). The total PHQ-9 score ranged from 0 to 27 with a higher score indicating a higher level of depressive symptoms. A cutoff score of 10, indicating moderate depressive symptoms, was used to screen the clinical level of depressive symptoms (39). Cronbach's alpha of the PHQ-9 was 0.88 in the present study.

### Potential Associated Factors

The potential associated factors were measured by nine items. Three items were designed to measure the experience of COVID-19, including a history of quarantine for observation or treatment, family members or friends dying of COVID-19, and family members or friends being cured of COVID-19. Four items were designed to measure the status of social support, including the frequency of seeking help when facing insurmountable difficulties, the number of relatives or friends providing support and assistance, the level of support and assistance that students received from family, and the relationship that students had with their parents at home. Another two items were separately designed to measure the daily time spent on electronic devices except for online learning and the feeling in the face of returning to school. Furthermore, if students felt anxious because of returning to school, an additional multiple-choice item was administered to ascertain the specific reason.

### Statistical Analysis

Data analyses were performed using SPSS version 20.0 (SPSS Inc., Chicago, IL). The prevalence of depressive symptoms was calculated based on a cutoff score of 10 and reported as the percentage of cases. Descriptive statistics expressed as frequencies and percentages were computed for all variables. Binary logistic regression analyses were conducted to explore the factors associated with depressive symptoms. All statistically significant variables in the univariate analyses were adjusted in the multivariable analyses using the “enter” method. Unadjusted and adjusted odds ratios (ORs) and 95% confidence intervals (CIs) were computed in the regression models. The Hosmer–Lemeshow test was simultaneously used to measure goodness of model fit, and the criterion of an appropriate logistic regression model was a *p* value greater than 0.05 (42). All hypothesis tests were two-tailed, and the significance level was set at α = 0.05.

## Results

As shown in Table 1, most participants were female (71.4%), of Han ethnicity (87.6%), and from urban areas (66.3%). Freshman, sophomore, and junior students accounted for 38.9, 35.7, and 25.4% of the study population, respectively. Approximately half of the participants were only children (54.0%) in their families. A total of 1,486 students had moderate-to-severe depressive symptoms, which accounted for 15.8% of the participants.

**Table 1:** Binary logistic regression analyses on the factors associated with depressive symptoms among 9383 college students in Wuhan, China during the normalization stage of COVID-19 prevention and control.

**Variables**	***N*** **(%)**	**Depressive Symptoms (%)**	**Unadjusted OR (95%CI) ^a^**	***p***-**value**	**Adjusted OR (95%CI) ^a^**	***p***-**value**
**Sex**						
Male	2,685 (28.6)	424 (15.8)	Ref.			
Female	6,698 (71.4)	1,062 (15.9)	1.00 (0.89 1.14)	0.939		
**College year**						
Freshman	3,654 (38.9)	560 (15.3)	Ref.			
Sophomore	3,348 (35.7)	551 (16.5)	0.97 (0.84 1.12)	0.656		
Junior	2,381 (25.4)	375 (15.7)	1.05 (0.91 1.22)	0.473		
**Ethnicity**						
Han	8,218 (87.6)	1,303 (15.9)	Ref.			
Minorities	1,165 (12.4)	183 (15.7)	0.99 (0.84 1.17)	0.897		
**Residence**						
Urban	6,220 (66.3)	926 (14.9)	Ref.		Ref.	
Rural	3,163 (33.7)	560 (17.7)	1.23 (1.10 1.38)	<0.001	1.11 (0.96 1.28)	0.154
**Only child**						
Yes	5,071 (54.0)	740 (14.6)	Ref.		Ref.	
No	4,312 (46.0)	746 (17.3)	1.22 (1.10 1.37)	<0.001	1.06 (0.92 1.21)	0.430
**Being quarantined for observation or treatment because of confirmed or suspected COVID-19**						
No	9,167 (97.7)	1,431 (15.6)	Ref.		Ref.	
Yes	216 (2.3)	55 (25.5)	1.85 (1.35 2.52)	<0.001	1.52 (1.08 2.15)	0.017
**Family members or friends dying of COVID-19**						
No	9,331 (99.4)	1,469 (15.7)	Ref.		Ref.	
Yes	52 (0.6)	17 (32.7)	2.60(1.45 4.65)	<0.001	2.35 (1.21 4.57)	0.012
**Family members or friends cured of COVID-19**						
No	9,193 (98.0)	1,449 (15.8)	Ref.			
Yes	190 (2.0)	37 (19.5)	1.29 (0.90 1.86)	0.167		
**Seeking help from others when facing insurmountable difficulties**						
Frequently	2,539 (27.1)	264 (10.4)	Ref.		Ref.	
Sometimes	3,860 (41.1)	505 (13.1)	1.30 (1.11 1.53)	0.001	1.06 (0.90 1.26)	0.473
Rarely	2,347 (25.0)	528 (22.5)	2.50 (2.13 2.94)	<0.001	1.57 (1.32 1.88)	<0.001
Never	637 (6.8)	187 (29.4)	3.58 (2.89 4.43)	<0.001	1.71 (1.35 2.18)	<0.001
**Number of relatives or friends who could provide support and assistance**						
≥6	2,391 (25.5)	195 (8.2)	Ref.		Ref.	
3~5	4,964 (52.9)	691 (13.9)	1.82 (1.54 2.15)	<0.001	1.45 (1.22 1.73)	<0.001
1~2	1,923 (20.5)	556 (28.9)	4.58 (3.84 5.47)	<0.001	2.54 (2.09 3.08)	<0.001
0	105 (1.1)	44 (41.9)	8.12 (5.37 12.29)	<0.001	2.19 (1.35 3.55)	0.002
**Support from family in the past month**						
Full	5,507 (58.7)	546 (9.9)	Ref.		Ref.	
General	3,322 (35.4)	733 (22.1)	2.57 (2.28 2.90)	<0.001	1.58 (1.37 1.81)	<0.001
Little	456 (4.9)	165 (36.2)	5.15 (4.17 6.36)	<0.001	1.97 (1.54 2.53)	<0.001
None	98 (1.0)	42 (42.9)	6.82 (4.52 10.27)	<0.001	2.13 (1.30 3.48)	0.003
**Relationship with parents at home**						
Harmonious	3,350 (35.7)	283 (8.4)	Ref.		Ref.	
Normal	5,636 (60.1)	1,025 (18.2)	2.41 (2.10 2.77)	<0.001	1.62 (1.39 1.89)	<0.001
Indifferent	308 (3.3)	137 (44.5)	8.68 (6.72 11.21)	<0.001	3.36 (2.51 4.51)	<0.001
Hostile	89 (0.9)	41 (46.1)	9.26 (6.00 14.29)	<0.001	5.45 (3.36 8.84)	<0.001
**Daily time spent on electronic devices except for online learning (h)**						
<2	299 (3.2)	27 (9.0)	Ref.		Ref.	
2~4	2,214 (23.6)	221 (10.0)	1.12 (0.74 1.70)	0.605	1.11 (0.71 1.73)	0.641
4~6	3,769 (40.2)	518 (13.7)	1.61 (1.07 2.41)	0.022	1.43 (0.93 2.20)	0.102
6~8	2,196 (23.4)	446 (20.3)	2.57 (1.71 3.86)	<0.001	2.10 (1.36 3.25)	0.001
≥8	905 (9.6)	274 (30.3)	4.37 (2.87 6.66)	<0.001	3.25 (2.08 5.08)	<0.001
**Feeling in the face of returning to school**						
Expectant	2,885 (30.7)	392 (13.6)	Ref.		Ref.	
Calm	5,306 (56.5)	708 (13.1)	0.98 (0.86 1.12)	0.757	0.99 (0.86 1.14)	0.886
Anxious	1,192 (12.7)	383 (32.4)	3.05 (2.59 3.58)	<0.001	2.53 (2.13 3.01)	<0.001

a *OR, odds ratio; CI, confidence interval; the adjusted OR and 95% CI were computed using multivariable non-conditional logistic regression analyses*.

Regarding the experience of the pandemic, 216 (2.3%) students were quarantined for observation or treatment because of confirmed or suspected COVID-19, 52 (0.6%) reported that their family members or friends had died of COVID-19, and 190 (2.0%) reported that their family members or friends had been cured of COVID-19.

Approximately one third of students rarely (25.0%) or never (6.8%) sought help from others when facing insurmountable difficulties. Nearly 80% of students (78.4%) had more than 3~5 relatives or friends who could provide support and assistance. In the past month, more than half of students (58.7%) felt fully supported by family, and 35.4, 4.9, and 1.0% felt that they received general, little, and no support from family, respectively. Approximately one third of students (35.7%) and 60.1% separately reported harmonious and normal relationships with parents at home although fewer students reported indifferent (3.3%) and hostile (0.9%) relationships with parents at home. Moreover, most students (67.0%) spent <6 h daily on electronic devices except for online learning, 23.4% of students spent 6–8 h, and 9.6% of students spent more than 8 h.

**Figure 1:**
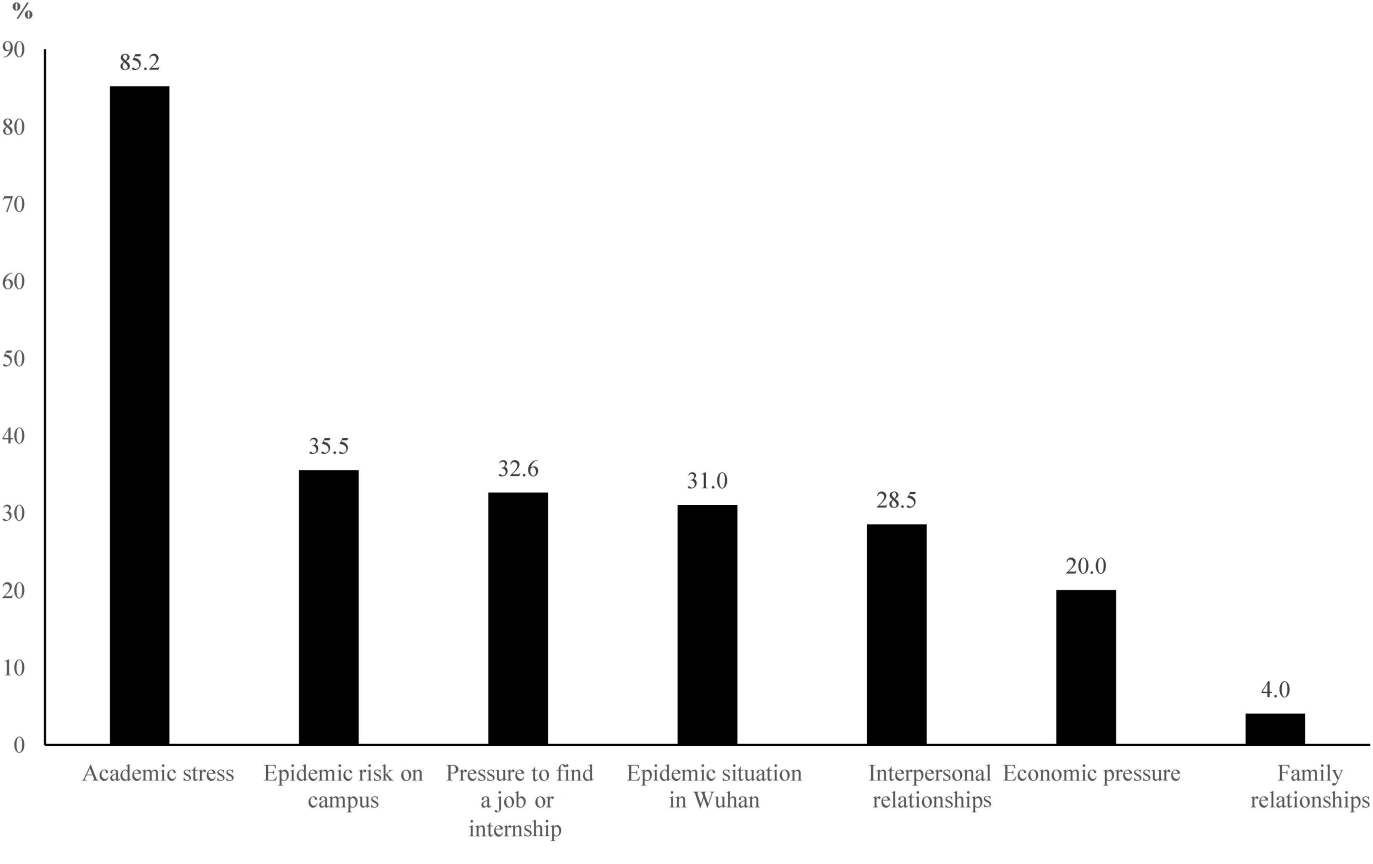
The reasons for anxiety in the face of returning to school among college students in Wuhan, China during the normalization stage of COVID-19 prevention and control (*n* = 1,192).

A total of 1,192 (12.7%) students felt anxious in the face of returning to school. Additionally, as shown in Figure 1, the primary reason for their anxiety was academic stress (85.2%), followed by the epidemic risk on campus (35.5%), pressure to find a job or internship (32.6%), and the epidemic situation in Wuhan (31.0%).

The statistical magnitude (χ^2^ = 8.99, *P* = 0.343 > 0.05) of the Hosmer–Lemeshow test showed that the binary logistic model was appropriate. As presented in Table 1, the experience of being quarantined for observation or treatment for confirmed or suspected COVID-19 was associated with an increased risk of depressive symptoms (OR, 1.52; 95% CI, 1.08 2.15; *P* = 0.017). Having family members or friends dying of COVID-19 was also associated with an increased risk of depressive symptoms. (OR, 2.35; 95% CI, 1.21 4.57; *P* = 0.012).

Students who rarely (OR, 1.57; 95% CI, 1.32 1.88; *P* < 0.001) or never (OR, 1.71; 95% CI, 1.35 2.18; *P* < 0.001) sought help from others when facing insurmountable difficulties were at a higher risk of depressive symptoms. Students with fewer relatives or friends who could provide support and assistance were more likely to have depressive symptoms (3 ~ 5 vs. ≥ 6: OR, 1.45; 95% CI, 1.22 1.73; *P* < 0.001; 1 ~ 2 vs. ≥ 6: OR, 2.54; 95% CI, 2.09 3.08; *P* < 0.001; 0 vs. ≥ 6: OR, 2.19; 95% CI, 1.35 3.55; *P* = 0.002). The risk of depressive symptoms increased as the level of support from family in the past month decreased (general vs. full: OR, 1.58; 95% CI, 1.37 1.81; *P* < 0.001; little vs. full: OR, 1.97; 95% CI, 1.54 2.53; *P* < 0.001; none vs. full: OR, 2.13; 95% CI, 1.30 3.48; *P* = 0.003). Students who reported a worse relationship with parents at home had a higher risk of depressive symptoms (normal vs. harmonious: OR, 1.62; 95% CI, 1.39 1.89; *P* < 0.001; indifferent vs. harmonious: OR, 3.36; 95% CI, 2.51 4.51; *P* < 0.001; hostile vs. harmonious: OR, 5.45; 95% CI, 3.36 8.84; *P* < 0.001).

A longer time that students spent daily on electronic devices except for online learning was associated with a higher risk of depressive symptoms (6 ~ 8 vs. <2 h: OR, 2.10; 95% CI, 1.36 3.25; *P* = 0.001; ≥8 vs. <2 h: OR, 3.25; 95% CI, 2.08 5.08; *P* < 0.001). Feeling anxious in the face of returning to school was associated with a higher likelihood of depressive symptoms compared with feeling expectant (OR, 2.53; 95% CI, 2.13 3.01; *P* < 0.001).

## Discussion

The prevalence of depressive symptoms was 15.8% among students attending university in Wuhan during the normalization stage of COVID-19 prevention and control in China. The experience of being quarantined for observation or treatment, family members or friends dying of COVID-19, rarely or never seeking help from others, fewer supportive relatives or friends, less support from family in the past month, a worse relationship with parents at home, a longer time daily spent on electronic devices except for online learning, and feeling anxious in the face of returning to school were associated with a higher risk of depressive symptoms. Academic stress and the risk of the epidemic were the main reasons for students feeling anxious in the face of returning to school.

Based on the PHQ-9 with the same cutoff score of 10, the prevalence of depressive symptoms (15.8%) in the present study was lower than that among college students from other countries, such as Spain (65.8%), Pakistan (45.0%), Ukraine (31.7%), and Switzerland (27.2%) during the COVID-19 epidemic (19–22). These differences between studies might be related to differences in the composition of the sample, cultures, regions, education systems, or survey time nodes as well as different impacts of the pandemic (22, 43). For example, approximately one quarter of students (22.9%) lived in an environment with an infected person in the Spanish sample (19), and more than 20% of students (21.8%) had significant others diagnosed with COVID-19 in the Pakistan sample (21). These situations may lead to a higher level of depressive symptoms among students. However, only 2.6% of students had family members or friends diagnosed with COVID-19 in the present sample. Moreover, almost all of the above surveys were conducted during the lockdown stage of the pandemic, and our survey was conducted during the normalization stage of COVID-19 prevention and control, which may also lead to the varying prevalence of depressive symptoms among the existing literature.

In addition, with the same measure and cutoff score, the prevalence of depressive symptoms in the current study was higher than that in other Chinese regions, such as Guangdong (4.2%) and Fujian (7.7%) Provinces as well as the cities Chengdu and Chongqing (9.0%) during the peak or containment stage of the COVID-19 epidemic (13, 17, 18). To better compare the prevalence of depressive symptoms in our sample with that (21.1%) in a previous Chinese national sample during the COVID-19 outbreak (15), we calculated the prevalence based on a cutoff score of 7, and a similar higher prevalence (52.4%) was found. Regarding experiences in Wuhan when COVID-19 first broke out, many students were urged to centralize or quarantine at home for 2 weeks after they returned home for the winter holiday. This unique experience may have led them to feel greater discrimination in various ways, such as social avoidance and abusive expressions (26, 44). Otherwise, compared with students in other regions, those attending colleges and universities in Wuhan experienced longer online learning and social isolation durations and may have felt more loneliness, spent more time on electronic devices, and engaged in less physical exercise (12, 13, 22). These factors might lead to a higher level of depressive symptoms. Furthermore, our results also imply that the pandemic had persistent negative psychological effects on Chinese college students even after it had been almost completely under control in China.

Therefore, students with moderate-to-severe depressive symptoms, who might need more professional psychological help, should be given more attention (45). However, during the COVID-19 pandemic, the rate of seeking psychological help was very low (0.6%) among college students (46). Meanwhile, we also found that 6.8% of students never sought help from others when facing insurmountable difficulties in the present study. However, for difficulties with respect to face-to-face contact, different forms of online mental health services were provided for the public in China, including online mental health education programs and online psychological counseling services (47), and at each Chinese college or university, the psychological health counseling center also provided online counseling services for students. Therefore, relevant departments should enhance promotion and education strategies to improve student's awareness of these public and scholastic sources of psychological support, reduce negative attitudes toward psychological counseling, and promote positive psychological help-seeking behavior. Additionally, this finding also suggests that the relevant departments should strengthen the mental health screening among back-to-school students in Wuhan, which may be beneficial to prevent psychological crisis events.

Consistent with previous studies (24–26), we found that the experience of being quarantined for observation or treatment was a risk factor for depressive symptoms among students. In the period of quarantine, individuals might experience increased negative emotions, such as fear, helplessness, sadness, stress, anxiety, and uncertainty, which contribute to the development of depressive symptoms (3, 4, 37). Moreover, these psychological impacts might last for a long time and lead to further deterioration of student's physical and psychological health status (3, 4). To address these issues, relevant departments should provide essential psychological support for this group. Furthermore, previous studies showed that having relatives or friends with COVID-19 was a risk factor for depressive symptoms (15, 24). In our study, having relatives or friends dying of COVID-19 was found to be a risk factor for depressive symptoms, indicating that these students should be given more attention. During the pandemic, the remains of all COVID-19 patients were uniformly disposed of by the government, preventing bereaved individuals from bidding farewell to their loved ones and performing funeral rituals to cope with their grief (48), which may have led to strong feelings of regret and self-blame for the bereaved individuals and increased the risk of depressive symptoms (49). Some psychological interventions, such as grief counseling and online sacramental ceremonies, should be implemented for this group to prevent negative emotional difficulties (50, 51).

The present results emphasize the importance of strong social support to prevent mental health disorders, which has been previously demonstrated (22, 30, 31). Positive social support can not only buffer the effect of uncertainty and stress related to the pandemic on depressive symptoms (30, 32), but also increase the feeling of social connectedness, which could protect students against depressive symptoms (12). However, never or rarely seeking help from others or having fewer supportive relatives or friends may cause students to feel less connected with others and lead to a higher level of depressive symptoms. Even though the company of parents was a primary support source during the pandemic, which played an important role in reducing the risk of depressive symptoms among students (31, 52), the quality of company might have had a further impact on their mental health (53). We found that less support from family and disharmonious relationships with parents were risk factors for depressive symptoms among students, which was in line with previous findings (12, 54, 55). The results provided us with some insight into preventing depressive symptoms among college students during a pandemic event. First, after returning to school, relevant departments should be concerned about the subsequent effect of negative family support on student's mental health and provide essential assistance to them. Second, when students were quarantined or had to learn at home during a pandemic, the content of psychosocial services should include guidance for improving the parent–child relationship and psychological support for parents. Third, relevant departments at colleges can organize online intervention activities to encourage students to connect with peers and further decrease the feeling of loneliness and depressive symptoms. In addition, we found that students who spent more time daily on electronic devices, except for online learning, had a higher risk of depressive symptoms. These students may have more frequently engaged in social comparisons with others (56); had limited opportunities for face-to-face contact with parents, relatives, and friends; and experienced less social support, more emptiness, and low self-worth (57), all of which may increase the risk of depressive symptoms.

Anxiety and depression are two issues that received substantially much more attention during the pandemic, both of which are widely known to have reciprocal positive correlations (37, 58) and can predict each other (59, 60). In the present study, we found that 1,192 (12.7%) students felt anxious when facing returning to school and experienced more depressive symptoms than expected. Regarding anxiety, only one third of students felt anxious about the risk of the pandemic, and more than 80% of students felt anxious about academic stress. The results implied that online learning might increase academic stress and indeed cause a negative impact on mental health among students (12, 61). After students returned to school, relevant departments should provide more academic support, such as academic guidance from teachers or trustworthy peers and lectures on learning methods or time management. Considering that the stage of epidemic prevention and control can last for a long time, colleges should regularly disclose prevention and control measures through official channels, which would be beneficial to decreasing student's feeling of uncertainty and, thus, reducing their anxiety and depression (32, 62). Regarding the pressure of finding a job, the employment guidance department should carry out relevant thematic educational and consultation activities for these students. Meanwhile, more employment information should also be summarized and published for students. For economic pressure, relevant departments should provide suitable financial aid for students according to their needs. For issues related to interpersonal relationships or family relationships, the counseling service center could design professional psychological activities, such as group counseling, lectures, and curricula, to support these students. Furthermore, colleges could also develop evidence-based intervention programs, especially those delivered by online technology, such as mindfulness meditation and cognitive behavioral therapy, to reduce student's anxiety and depression (63, 64).

This study has some limitations. First, the cross-sectional design complicates causal inference. Second, a nonrandom sampling method was used, and the students were from the same college in Wuhan; therefore, the sample was not fully representative of all Chinese college students although these students were from all regions of China. Third, the status of depressive symptoms was determined by an online questionnaire rather than a clinical diagnosis. Fourth, some mediation or moderation effects were not considered in our study. Large-scale studies with longitudinal designs and randomized sampling methods as well as clinical diagnoses for depressive symptoms should be conducted in the future.

## Conclusion

In summary, 15.8% of non-graduating undergraduate students in Wuhan suffered from moderate-to-severe depressive symptoms during the normalization stage of COVID-19 prevention and control in China. Students with negative experiences of COVID-19, less support from family and friends, longer time spent daily on electronic devices except for online learning and feeling anxious in the face of returning to school had a higher likelihood of depressive symptoms. Academic stress and concern about epidemic control were the main reasons for student's anxiety. Based on these findings, professional psychological support and assistance are urgently needed for these students with a high risk of depressive symptoms. We recommend that targeted psychological intervention measures should also be developed for college students to encourage them to proactively seek professional psychological help and improve their mental health during the normalization stage of COVID-19 prevention and control in China.

## Data Availability Statement

The raw data supporting the conclusions of this article will be made available by the authors, without undue reservation.

## Ethics Statement

The studies involving human participants were reviewed and approved by the Institutional Review Board of Zhongnan University of Economics and Law. The patients/participants provided their written informed consent to participate in this study.

## Author Contributions

HJ supervised the study design and statistical analyses, and provided substantial comments to improve the draft. ZY and MG conceptualized of the study and designed the questionnaire. JY, YW, ZY, and MG sorted out the data. JY, YW, and XT conducted the literature search and data analyses. JY drafted the manuscript. YW revised the language. All authors reviewed and approved the submitted version.

## Funding

This study was approved by the Fundamental Research Funds for the Central Universities, Zhongnan University of Economics and Law (2722020SQY07).

## Conflict of Interest

The authors declare that the research was conducted in the absence of any commercial or financial relationships that could be construed as a potential conflict of interest.

## Publisher's Note

All claims expressed in this article are solely those of the authors and do not necessarily represent those of their affiliated organizations, or those of the publisher, the editors and the reviewers. Any product that may be evaluated in this article, or claim that may be made by its manufacturer, is not guaranteed or endorsed by the publisher.
